# Prescribers’ perspectives on including reason for use information on prescriptions and medication labels: a qualitative thematic analysis

**DOI:** 10.1186/s12913-021-06103-1

**Published:** 2021-01-26

**Authors:** Colin Whaley, Ashley Bancsi, Joanne Man-Wai Ho, Catherine M. Burns, Kelly Grindrod

**Affiliations:** 1 University of Waterloo School of Pharmacy, Health Sciences Campus, 200 University Ave West, Waterloo, ON N2L 3G1 Canada; 2 Michael G. DeGroote School of Medicine, McMaster University, 1280 Main Street West, Hamilton, ON L8S 4L8 Canada; 3 Divisions of Geriatric Medicine & Clinical Pharmacology and Toxicology, Department of Medicine, McMaster University, 1280 Main Street West, Hamilton, ON L8S 4L8 Canada; 4 Schlegel-UW Research Institute for Aging, 250 Laurelwood Drive, Waterloo, N2J 0E2 ON Canada; 5 GeriMedRisk, 250 Laurelwood Drive, Waterloo, N2J 0E2 ON Canada; 6 Department of Systems Design Engineering, Faculty of Engineering, University of Waterloo, 200 University Ave West, Waterloo, ON N2L 3G1 Canada

## Abstract

**Background:**

The indication for prescribing a particular medication, or its reason for use (RFU) is a crucial piece of information for all those involved in the circle of care. Research has shown that sharing RFU information with physicians, pharmacists and patients improves patient safety and patient adherence, however RFU is rarely added on prescriptions by prescribers or on medication labels for patients to reference.

**Methods:**

Qualitative interviews were conducted with 20 prescribers in Southern Ontario, Canada, to learn prescribers’ current attitudes on the addition of RFU on prescriptions and medication labels. A trained interviewer used a semi-structured interview guide for each interview. The interviews explored how the sharing of RFU information would impact prescribers’ workflows and practices. Interviews were recorded, transcribed and thematically coded.

**Results:**

The analysis yielded four main themes: Current Practice, Future Practice, Changing Culture, and Collaboration. Most of the prescribers interviewed do not currently add RFU to prescriptions. Prescribers were open to sharing RFU with colleagues via a regional database but wanted the ability to provide context for the prescribed medication within the system. Many prescribers were wary of the impact of adding RFU on their workflow but felt it could save time by avoiding clarifying questions from pharmacists. Increased interprofessional collaboration, increased patient understanding of prescribed medications, avoiding guesswork when determining indications and decreased misinterpretation regarding RFU were cited by most prescribers as benefits to including RFU information.

**Conclusions:**

Prescribers were generally open to sharing RFU and clearly identified the benefits to pharmacists and patients if added. Critically, they also identified benefits to their own practices. These results can be used to guide the implementation of future initiatives to promote the sharing of RFU in healthcare teams.

**Supplementary Information:**

The online version contains supplementary material available at 10.1186/s12913-021-06103-1.

## Background

**Fig. 1:**
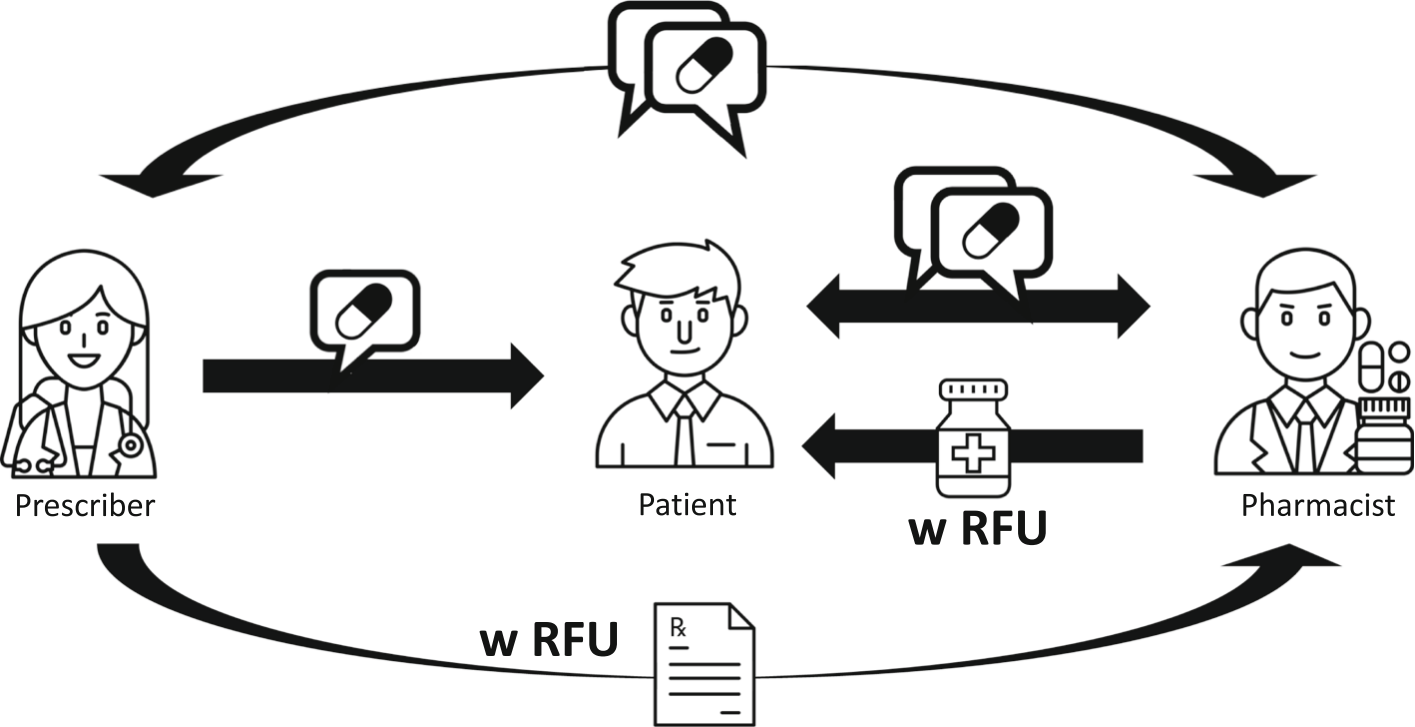
Current RFU information flow. Speech bubbles denote verbal communication, either in person or on the phone. Prescribers (left) verbally explain RFU to patients (middle), who are expected to relay this information to pharmacists (right). Image credits: doctor, pharmacist, office worker by Amethyst Studio; talk by Markus; pill by Nithinan Tatah, formula by faisalovers, medication by BTL Bay, all from thenounproject.com

Many North Americans use medications to treat illnesses and manage their health. In Canada, 66% and in the United States, 69% of adults aged 40–79 used at least one prescription medication in the last 30 days as found by the US National Health and Nutrition Examination Survey (2015–2016) and the Canadian Health Measures Survey (2016–2017), respectively [1]. Prescription medications rarely include the reason they are prescribed, called reason for use (RFU) [2]. Existing literature has called for RFU to be included in two places: on the prescription sent to the pharmacy and on the medication label, Fig. 1. The addition of RFU to prescriptions can have positive impacts on medication safety, patient understanding of their medications, and patient adherence to agreed-upon treatment regimens, by helping pharmacists understand medications when dispensing them [3, 4]. In an analysis of more than 4.3 million outpatient prescriptions issued between 2011 and 2015 from a major academic medical centre in Illinois, only 7% of prescriptions included the RFU [2].

Despite efforts by prescribers to educate patients about their medications, patients may still lack a clear understanding of why a particular medication was prescribed, which is associated with an increased number of adverse drug events and decreased patient adherence [5, 6]. In the United States, Persell et al. showed that patients belonging to populations associated with increased morbidity, namely those who are older, less educated, or black, are less likely to know their medications’ RFU [7, 8]. However, pharmacists who have access to the RFU catch more medication errors, reduce unnecessary contact with prescribers, and reinforce physician education of patients [9–11]. Current understanding for why RFU is not currently added to prescriptions lies in the additional effort required to add RFU to prescriptions, and concerns about privacy, rather than any unintended effects on patient safety [2, 4]. Despite these and other studies showing the value of adding RFU to prescriptions, guidelines from physicians groups encouraging the clear communication of medication information, as well as support from various healthcare advocacy groups, prescribers must ultimately choose to add RFU to the prescriptions they write [12–15]. Existing literature and patient safety organizations have proposed the addition of RFU on two locations: on the prescriptions sent by prescribers to pharmacists, and on medication labels, as seen in Fig. 1 [4, 12, 13, 16, 17]. Adding RFU to the prescription would serve to communicate the RFU directly to the pharmacist, whereas adding it to the medication label would allow patients to better understand the medications they are taking [18].

Much of the literature has focused on technological ways of making the addition of RFU more straightforward, and engaging high-level stakeholders in the value of adding RFU, however no studies have directly asked prescribers for their perspective on the addition of RFU on prescriptions [4, 18]. From an implementation perspective, it is critical to understand its potential value for prescribers relative to the perceived impact on workflow [19]. The objective of this paper was to explore physician perspectives on writing medication RFUs on prescriptions and medication labels.

## Methods

We conducted semi-structured interviews with 20 prescribers (2 nurse practitioners, 18 physicians) in Southern Ontario, Canada between June and August 2018. The interviews were conducted as a part of a larger study evaluating how pharmacists, prescribers and patients currently communicate RFU, how policies around RFU sharing may impact healthcare teams, and clinicians’ perceptions on sharing RFU with their colleagues and patients. Semi-structured interviews were used, as they allowed the flexibility to follow-up on key concepts mentioned by participants [20]. Ethics approval was received by a University of Waterloo research ethics committee (ORE# 31591).

Prescribers in AB and KG’s networks and were asked to participate in this study. Upon completion, they were also asked if they knew any colleagues who may also be willing to participate in the study. This snowball sampling method was used to gather additional participants. Prescribers were interviewed at a time and location of their choosing, often over the phone or at their clinic. A $150 CAD honourarium was provided in appreciation for their time. Information on participants’ demographics, profession, specialty and years of practice was collected, and can be found in Table 1. Additional aggregated demographic information can be found in Table 2.

**Table 1:** Demographic profile of participants

ID	Gender	Type	Specialty	Years in Practice
Prescriber 01	Female	Nurse practitioner	–	Unknown
Prescriber 02	Female	Nurse practitioner	–	9
Prescriber 03	Female	Physician	Family medicine	20
Prescriber 04	Female	Physician	Family medicine	32
Prescriber 05	Male	Physician	Family medicine	30
Prescriber 06	Male	Physician	Family medicine	8
Prescriber 07	Male	Physician	Family medicine	33
Prescriber 08	Male	Physician	Family medicine	1
Prescriber 09	Male	Physician	Emergency medicine	4
Prescriber 10	Male	Physician	Family medicine	29
Prescriber 11	Female	Physician	Emergency medicine	5
Prescriber 12	Male	Physician	Emergency medicine	4
Prescriber 13	Male	Physician	Emergency medicine	4
Prescriber 14	Female	Physician	Family medicine	7
Prescriber 15	Male	Physician	Emergency medicine	5
Prescriber 16	Male	Physician	Family medicine	2
Prescriber 17	Female	Physician	Family medicine	2
Prescriber 18	Female	Physician	Family medicine	2
Prescriber 19	Male	Physician	Anesthesiology	7
Prescriber 20	Male	Physician	Family medicine	2

**Table 2:** Practice details for participants

Recruitment method	Personal network: 7 Snowball sampling: 13
**Practice type**	Community health centre: 4 Family health team: 4 Hospital: 7 Independent practice: 5
**Practice Location**	Southwestern Ontario: 14 Greater Toronto Area: 6
**Academic appointment**	Adjunct appointment: 4 Full appointment: 3 None: 13

### Data collection

Interviews were conducted by one pharmacy and one systems design engineering researcher using a semi-structured interview guide jointly developed by the engineering and pharmacy teams (Appendix 1). Feedback from prescribers and patients was sought in when developing these questions. Specifically, we used a qualitative approach to ask prescribers the following:

- how their practice and clinical workflow would be impacted by being required to add RFU onto prescriptions;
- how sharing RFU information on prescriptions would impact their relationships with other prescribers, pharmacists and patients, and;
- the perceived impact of having RFU information shared on patient medication labels.

Interviews were recorded and transcribed. In the interviews, prescribers were asked about their current clinical workflow and how RFU fits into it, changes to workflow as a result of adding RFU, and how adding RFU could impact professional relationships with clinicians and patients.

### Data analysis

Transcripts were stored and analyzed using NVivo 12 for Mac [21]. Thematic analysis allowed for prevalent participants’ opinions to be expressed while preserving unique perspectives [20]. Iterative coding allowed themes to develop over the course of reviewing the interviews and ensured that the final themes were aligned with what participants said.

Two members of the pharmacy research team (CW, KG) coded the first three interviews independently and generated a list of codes. Differences in coding were reviewed for each interview, and discrepancies resolved code-by-code. Both CW and KG reviewed the remaining interviews and met periodically to review codes and resolve discrepancies, by discussing the rationale for particular codes. Through this process, the codebook was updated as new codes emerged, upon the agreement of both researchers. CW and KG assembled the quotes into larger themes, and the quotes were synthesized into a Framework Matrix using NVivo 11 for Windows. Quotes in the Matrix were reviewed, and representative and divergent quotes were selected for our results. The final analysis was reviewed by a physician researcher to provide additional background and context. Memos were periodically written during the coding process, to facilitate theme generation and refinement. The engineering team separately analyzed the data, and developed a model of prescriber decision making, which is published elsewhere [22]. Inductive thematic saturation was reached on the basis of no new codes being observed in the data [23]. While preparing the manuscript, the Standards for Reporting Qualitative Research (SRQR) were followed (Appendix 2) [24].

## Results

Twenty prescribers (18 physicians, 2 nurse practitioner) were interviewed. Participants included the following specialties: 12 family medicine, 5 emergency medicine, and 1 anesthesiology. Additional information can be found in Table 1 and aggregated demographic information can be found in Table 2. While gender was not a focus of this analysis, there did not appear to be a clear difference between the opinions of prescribers of different genders.

Most of the prescribers acknowledged that adding RFU onto their prescriptions would take additional time and result in some additional workload. However, the prescribers also generally acknowledged that there would be benefits to their clinical practice. These aspects are captured in the following four themes: current practice; future practice; changing culture; and collaboration.

### Theme 1: current Practice

Throughout the interviews, prescribers were invited to comment on their current practice with respect to RFU, and how they thought pharmacists were currently determining and using RFU to dispense the prescribed medications. More than three quarters of the prescribers interviewed indicated that they do not routinely add RFU onto prescriptions, with some exceptions. For example, one prescriber indicated that they add RFU in two situations:

Mainly [adding RFU] for PRNs, more than likely for medications that might be a short-term use for a new indication. That might be the time you might [add the RFU]... or particularly with an older person. [Prescriber 04, female]

When asked to speculate on how pharmacists currently obtain RFU, the prescribers believed that pharmacists ask the patients, or guess. However, they repeatedly acknowledged that patients can be unreliable sources of information. The prescribers themselves acknowledged the dissonance between their expectation that pharmacists receive accurate RFU from patients, and their experiences with patients not understanding aspects of their own care:

[Pharmacists] might ask the patient. Patients might not always know, we know that. … But certainly there’s going to be a lot of confusion … I would imagine that most [pharmacists] have so much experience dealing with [prescribers] that they understand, probably, what’s going on, but that’s not a good explanation. [Prescriber 06, male]

This prescriber noted that pharmacists appear to be generally competent at using context and experience to determine the RFU. But as the prescriber noted, this is not a good replacement for clear interprofessional communication. While the prescribers generally believed pharmacists could benefit from the information, there was a concern that not all pharmacists would make good use of it:

Large pharmacies like [national pharmacy chain], I don't think patients get a lot of counseling 'cause I think they're turning a lot of prescriptions, whereas my experience with smaller, community pharmacists is that the patients get a lot more information, and they get some information to help them understand why they were prescribed that medication. But I don't think it happens consistently. [Prescriber 10, male]

### Theme 2: future Practice

Prescribers were asked what they thought of adding RFU into region-wide drug database, such as an electronic health record or a drug profile viewer. They were generally supportive of allowing their colleagues to access information as to why a medication was prescribed.

I don't know why [a medication’s RFU] should be [a secret]. I think [having it in a regional database] would be good. [Prescriber 2, female]

But they were quick to note that having the opportunity to add context to the prescription would be beneficial.

… having that free-form box would be useful because sometimes the diagnosis is not always clear, so you have room to say ‘viral [upper respiratory infection] ruled out otitis media versus strep’ something like that. [Prescriber 2, female]

The notion of saving time on call backs from pharmacists was cited by a number of prescribers as one of the greatest strengths of adding RFU, with the following quote reflecting the opinion of a number of prescribers:

…[S]ometimes pharmacists send us notes back asking for clarifications, so there'll be time saved in not having those faxes of clarification. [Prescriber 10, male]

More than two thirds of prescribers were in agreement that they did not need support in adding RFU to prescriptions, despite the increase in work. Specific suggestions for how to assist with the addition of RFU are presented in Table 3. Some prescribers mentioned that since they already knew the indication, the only difference from current prescribing practice would be writing down what they were already thinking.

**Table 3:** Help with documenting RFU

Need help adding RFU?	Number of prescribers	Examples provided
Yes	8	5: No specifics 2: Help from EMR (eg, template, autosuggest RFU based on medication) 1: Additional training
No	10	7: No specifics 2: Help from EMR (eg, template, autosuggest RFU based on medication) 1: reminders to add RFU from EMR
Depends	2	1: Depends on patient population (eg, memory clinic, falls clinic, pain clinic) 1: Depends on specialty

I don't know why [prescribers] would need help to document the reason for use, because [they’re] prescribing [the medication] for a reason. [Prescriber 9, male]

However, many prescribers were wary of the impact that adding this task would have on their overall workflow.

The only concern I would have, is that it will just take an extra few minutes. [Prescriber 14, female]

Most prescribers who expressed concern about the impact of adding RFU on their workflow cited a similar issue: their concern with adding RFU is primarily in the cumulative time spent entering indications in their electronic medical records (EMRs), which could take away from time spent with patients. As one prescriber described:

…for meds that are being prescribed on a regular basis, once you put it in the EMR once, and you go to refill that med, it's gonna pop up, Reason for Use. So, it’s just a little bit more work up front. But I think it would be worth the effort, ultimately, at the end of the day given the benefit. [Prescriber 14, female]

However, one prescriber in particular felt that the time required of prescribers to add RFU to every prescription would be too great, and that a targeted approach in adding RFU only in situations where clarification would be of high utility was suggested. Situations cited by the prescribers interviewed as benefiting from the addition of RFU, as well as those where RFU should not be added are presented in Table 4.

**Table 4:** When to add RFU according to participants

Add RFU	Do not add RFU
• Older adults • Antibiotics for paediatric patients • Polypharmacy • Unusual dose or indication • Persons who need supports to take medications • As needed (PRN) medications • Medications for acute conditions	• Treatment for sensitive illnesses (e.g. sexually transmitted infections [STIs], psychiatric medications) • Adding RFU increases patient’s anxiety • Could affect patient’s likelihood of taking the medication • Off-label prescribing

### Theme 3: changing culture

Prescribers readily admitted that their training did not make them aware of the value of passing along RFU to pharmacists, but most could identify potential benefits when asked. While the goal of this study was not to influence prescriber behaviour, the mere act of asking prescribers how this information could be useful to pharmacists encouraged a number to consider adopting this more collaborative practice.

… I think it's a really good idea, and I never really thought about it and how important it is until today. May start doing it. [Prescriber 16, male]

In contrast to Prescriber 16, a different prescriber shared similar sentiment, but much more reservedly:

I guess the one thing that would be important before going forward with making something mandatory, I feel like a lot of doctors get nervous, or they don't like hearing about more mandatory stuff. And I understand, again, death by a thousand cuts, so it would be important to make sure that doctors, maybe the [medical associations], or whoever is in charge of doctors, weighed in and really felt comfortable and felt like they were on board with adding more responsibility to what doctors do. But I do think overall it sounds like a good idea. [Prescriber 11, female]

Prescribers may feel that some aspect of their autonomy in prescribing may be threatened if the inclusion of RFU is mandated without appropriate consultation. While many prescribers welcomed the additional set of eyes verifying that the prescribed medication was correct for the specific indication, others were concerned that routinely providing RFU to pharmacists may cause interprofessional conflict and could ultimately result in patients not taking their medications:

If the pharmacist disagrees [with my indication] then I really don't, as a physician, who has seen the patient and have spent some time with them, and have gone through their history; I would hate for that aspect of the care to be challenged and the patient not going on the medication… [Prescriber 19, male]

More than half of the prescribers interviewed also preferred the idea of a free text entry field for adding RFU, allowing them the flexibility to be precise in their documentation. Some of the prescribers mentioned the possibility of suggested RFU options within the EMR, integrated similar to an “autocomplete” function.

### Theme 4: collaboration

Prescribers were asked to comment on how listing RFU on prescriptions and medication containers would impact their relationships with other prescribers, pharmacists and patients.

With respect to their relationships with other prescribers, the prescribers interviewed noted that they would benefit from having ready access to RFU any time they had to understand why a patient was prescribed a particular medication (eg, after a visit to a different prescriber or when a prescriber takes over an existing practice). They also generally agreed that a regional electronic health record (EHR) or a medication label was the best and most convenient place to store this information for easy access. Few prescribers noted that their relationship with other prescribers would be impacted, but they generally agreed that ensuring other prescribers had access to this information would facilitate communication across the healthcare team:

I think it would enhance the relationship [between prescribers] because everyone is clearer and on board as to why you prescribed something for what reason. [Prescriber 10, male]

Less than one quarter of prescribers noted that adding RFU information could lead to “judgement” from their colleagues regarding the prescriptions they write:

… if I write an indication for use and [other physicians are] not used to that, they may question it. They may make judgements. [Prescriber 16, male]

Specific cases that were noted included if the other prescriber is not used to an indication being written in a particular way, or if the other prescriber lacks some contextual information surrounding the prescription.

Prescribers were also asked to comment on whether it would be useful to have access to a patient’s RFU for a medication when they did not prescribe it. They overwhelmingly had confidence in their ability to infer the prescriptions’ indications based on the medication list.

[When looking at a medication list] I probably don't need a lot of information [to determine RFU]. Cause I can look at the medication list and kinda understand what they're there for. [Prescriber 7, male]

With pharmacists, prescribers readily noted benefits such as an increase in bidirectional communication, supporting deprescribing, checking medication doses for a given indication to improve safety, improving counselling and patient education, and improving adherence. However, one prescriber expressed the following concern about the trustworthiness of pharmacies:

I mean, the only, my only concern about that would be if that information is being used for commercial purposes... You know, like say someone comes into a [national pharmacy chain] with [a sexually transmitted infection (STI)]. Is [national pharmacy chain] now going to target them with advertisements to get them to buy more condoms? [Prescriber 15, male]

This prescriber’s concern reflects the importance of ensuring all parties understand how this information may be used, and the need to support patient confidentiality when sharing information between clinicians.

With regards to relationships with patients, prescribers tended to be either positive or ambivalent about the potential impact of adding RFU on prescriptions. All of the prescribers noted that they explain to patients why a particular medication was prescribed. Some prescribers noted that benefits may not be experienced by all patients when adding RFU:

So, for some people, it may be a very helpful thing. It may help them to understand their medications a little bit better to make more informed users, I suppose. For others, it may just be not necessary information that they already knew. [Prescriber 4, female]

Others presented a view in light of their own experiences with the information patients know.

I feel like writing something down gives the patients something concrete, because I often end up writing things down for them anyway, so this way they have it already on their prescription. I feel like it gives them something concrete that they can go Google when they get home. [Prescriber 11, female]

The prescribers all acknowledged that patients have the right to know why they were prescribed a given medication, and generally agreed that providing written RFU would support patients in understanding their medications.

## Discussion

The prescribers interviewed were supportive of improving their communication with their pharmacist colleagues, especially because of the potential to support patient education and safety and to save time via reduced call backs about prescriptions. The potential increase in workload caused concern for many prescribers, but they did not feel they would need assistance in adding RFU to prescriptions. The few prescribers who currently add RFU to prescriptions tended to do so in limited circumstances, which seemed to be a palatable option for most prescribers. Prescribers’ speculation that pharmacists currently determine RFU by asking the patient, or by guessing is in agreement interviews that we conducted separately with pharmacists, and many participants empathized with the difficulty their pharmacy colleagues have in determining why a given medication was prescribed [25]. As evidenced by Prescriber 16, simply informing prescribers of the value of adding RFU to medications has the potential to encourage its routine addition on medication labels. Interprofessional training, such as through the use of pharmacy students and pharmacists to teach medical trainees the value of adding RFU to prescriptions may encourage this habit from the onset of prescribers’ careers [26–28].

Errors when entering medications into computerized provider order entry (CPOE) systems have been identified as a time where entering RFU could provide an additional opportunity to verify medication instruction [4, 6, 18]. Despite the concern about being “judged” when prescribing medications for certain indications, many prescribers were able to identify the benefits of ensuring pharmacists have access to RFU. One concern that emerged was the additional time that may be needed to enter RFU. A proposal for an “indications-based” system whereby RFU is entered first, followed by selecting a medication has been proposed to reduce the burden on prescribers [4, 17]. While this smaller-scale study yielded increased usability and time efficiency when using this indications-based system, studies demonstrating improved patient safety metrics (ie, reduced medication harm, increased adherence) would provide stronger evidence for the clinical value of adding RFU.

Patients may not always be good quality sources of information about their medications due to a variety of factors, including low health literacy and receiving poor information about their medications from clinicians [29–31]. The World Health Organization report *Medication Without Harm* (2017) referred to patients as often being “made to be passive recipients of medicines and not informed and empowered to play their part in making the process of medication safer.” [32] Prescribers 4 and 11 had a similar understanding of the utility of RFU to support patients understanding their medications, which is in line with previously identified patient preference [33, 34]. RFU can be a valuable tool for patient education, as mentioned by some of the prescribers interviewed, giving patients “something concrete” to take home after a health care appointment, [Prescriber 11, female] with the goal to make then “more informed users” [Prescriber 4, female]. Listing RFU on medication bottles could help patients have a greater understanding of their medications, potentially mitigating some risks of self-medication [35]. Notably, these benefits may be lessened by the adoption of paperless ePrescribing [25, 36, 37]. Additionally, there is a potential to add RFU to discharge prescriptions post-hospital stay, to assist with medication reconciliation [38]. Increasingly, institutions are recommending the addition of reason for use to discharge prescriptions, however additional work is needed in this area [39].

Participants were asked to reflect on how their relationships with other prescribers, pharmacists and their patients would be impacted by the addition of RFU. Few prescribers focused on these professional relationships, but they highlighted that adding RFU would likely lead to improved communication with other prescribers and pharmacists. When sharing RFU with pharmacists, some of the prescribers expressed concern about possible interprofessional conflict that could result if a pharmacist is critical of a prescription, which has been found to be a considerable source of stress for pharmacists [40]. While much of the RFU work has focused on time constrains, attention must be paid to the skills needed for interprofessional teamwork.

Throughout the interviews, the prescribers highlighted a number of cases where adding RFU would be particularly beneficial. If adding RFU is to be implemented in routine practice, phasing in its use beginning with select populations or medications may help to highlight the value of this practice to prescribers, help clarify expectations for the parties involved, and provide valuable information to pharmacists [25]. Adding RFU for medications being used for short-term use (eg, antibiotics) as stated by Prescriber 4 can facilitate medication reconciliation and deprescribing, encouraging medication lists to be up-to-date [18, 41].

In contrast to the guidance from some of the interviewed prescribers to not add RFU for medications prescribed off-label, Linsky et al. do recommend adding RFU for medications prescribed in this way [41]. Medications indicated for neurological and psychiatric illnesses have been found to be prescribed off-label frequently [42]. While RFU would be particularly valuable for off-label psychotropic medications, concerns about patient privacy were others to see the medication’s RFU could pose challenges relating to embarrassment for patients [25]. Future work will focus on developing guidelines for when RFU should be added to prescriptions and medication labels, and consulting with communities who live with sensitive illnesses to determine what they would see as best practice for sharing the RFU for their medications on prescriptions and medication labels.

### Limitations

This study sampled a limited number of prescribers in one geographic region in Canada, representing two types of prescribers (physicians and nurse practitioners) and three medical specialties (family medicine, emergency medicine, and anesthesiology). Some practitioners in the specialties interviewed (eg, emergency medicine, anesthesiology) may not provide routine follow-up with patients, resulting in limited context regarding the impact of pharmacy practice on medication therapy management. Additionally, we used snowball sampling to identify individuals interested in participating. We may have been referred to people who are generally more interested in promoting interprofessional collaboration, resulting in a sample biased towards sharing RFU. Finally, gaining perspectives from other clinicians (eg, dentists, optometrists), including those providing treatment in inpatient settings, and in other locations would enhance the transferability of these findings.

## Conclusion

This study highlights the aspects of RFU addition that may cause friction for its routine use, however it highlights a number of beliefs expressed by prescribers that should be used in implementation efforts. These results can be used to advocate for a staged rollout of RFU for select prescription classes/populations, and to support the implementation of new workflows such as indications-based prescribing. By keeping in mind the concerns of these prescribers as the push to routinely include RFU on prescriptions continues, increased interprofessional communication, increased patient understanding of their medications, and decreased harm from the use of medications can be achieved.

## Supplementary Information

**Additional file 1.** Interview Guide

**Additional file 2.** Checklist

## Data Availability

The data that support the findings of this study are available on request from the corresponding author C.W. The data are not publicly available due to the data containing information that could compromise participant privacy.

## Abbreviations

CPOE: computerized provider order entry
EHR: electronic health record
EMR: electronic medical record
PRN: *pro re nata* (as needed)
RFU: reason for use
SRQR: Standards for Reporting Qualitative Research
STI: sexually transmitted infection
