# Supplementary material for: Prescribers’ perspectives on including reason for use information on prescriptions and medication labels: a qualitative thematic analysis
Source: BMC Health Serv Res. 2021 Jan 26;21:89. doi: 10.1186/s12913-021-06103-1 (PMC7836591; doi:10.1186/s12913-021-06103-1)
Supplement: Supplementary file 1 — Additional file 1. Interview Guide [file 12913_2021_6103_MOESM1_ESM.pdf]

Appendix C: Interview Questions for Physicians and Nurse Practitioners

1. What is your gender? ___ Man ___ Woman ___ Non-binary

2. How many years have you been a physician or a nurse practitioner for? ______

3. Are you a family doctor? ____ Yes ____ No
   *If no, what is your medical specialty

4. What type of practice do you have?
   ____ Independent ____ Family health organization
   ____ Family health team Other: ______________________

5. What is the electronic medical record (EMR) system you use in your office?
   ____

6. Have you used other EMR systems, besides which you currently use?
   ____ Yes ____ No
   *If you do, which one(s)? __________________________
   *With which one do you feel most comfortable? ___________________
   * Why?

   We are going to talk about your experiences in the use of the current system.

1. How often do you enter reason to use information into the computer when you are writing your prescriptions?
   ____ Never ____ Rarely ____ Sometimes ____ Often ____ Always
   *If you do, who sees the reason for use information that you record?

2. When you were writing prescriptions, did you ever record the "reason for use" in your EMR?
   * If yes, how?
   * If no, why not?

3. Did you ever include it on the prescription?
   ____ Yes ____ No _____It depends (please explain)

4. Would you be willing to share reason for use with the pharmacist?
   ____ Yes ____ No

5. Do pharmacists need to know the “reason for use” for a prescription?
   ___ Yes ____ No _____It depends (please explain)
   * If yes, how?
   * If no, why not?

6. If the reason for use wasn’t given to a pharmacist, describe how you think a pharmacist identifies the “reason for use” of a medication?

7. Describe what you think a pharmacist could do with “reason for use” information?

8. Would you be willing to have the reason for use printed on the patient’s medication label?
   ___ Yes ____ No _____It depends (please explain)

9. Would you be willing to have the reason for use shared on a region-wide drug record such as *ClinicalConnect* or the *Ontario Drug Benefit Profile Viewer*?
   ___ Yes ____ No
   Why / why not?

10. If you knew other healthcare providers were going to see the reason for use for the medications you prescribe, would you write the “reason for use” in a different way than you would for your own records?
    ___ Yes ____ No
    If yes, how would it be different?

11. How beneficial would it be for you to share the "reason to use" with pharmacists and patients?
    ____ Not at all ____ A little ____ Somewhat ____ Quite a bit ____ A great deal
    If your answer were no, can you tell us why?
    If your answer was yes, what are those foreseeable advantages in each case (with other physicians, the pharmacist, and the patient)?

12. How strongly do you agree/disagree that patients should have access to “reason for use” for their prescriptions?
    ____ Strongly disagree ____ Disagree ____ Neutral ____ Agree ____ Strongly Agree

13. Do you have any concerns about adding “reason for use” to prescriptions?
    ___ Yes ____ No _____It depends (please explain)

14. Think back to the last month, when you were writing prescriptions for patients, how would they know the reason the drug was being prescribed? What do you tell your patients about the reason you are prescribing a medication?

We are going to talk about would happen if the "reason to use" it is added to the current system.

1. Imagine yourself in a situation where you are treating a new patient. You are looking at their medication list. How much information about the "reason for use" would you need to be able to renew their old medications?
   a. Diagnostic codes (e.g., ICD-9 codes)? ___ Yes ___ No ____ It depends
   b. Free form notes? ___ short answer (less than a sentence) ____ long answer (more than a sentence)
   c. Would physicians need help to document the "reason for use" on prescriptions? ____ Yes ____ No

2. What is the easiest way to share "reason for use" information with pharmacists and other physicians outside of your workplace?

3. If the "reason for use" was added to the prescription, what type of language should be used?
   ____ lay terms ____ medical terms

4. When a medication is initially prescribed, who should be responsible for translating the "reason to use" information into lay language for to the patient?
   ____ physician ____ pharmacist ___ both

5. How do you envision yourself including reason to use information on a prescription if it was mandatory?

6. How would it affect your workflow? What would be good? What would you worry about?

7. How would mandatory reason to use information influence your relationship with your patients? With other physicians? With pharmacists?

8. Do you have any final thoughts?

Could you suggest any colleagues who may be willing to speak with us?
